# Atrophy of the Teeth

**Published:** 1871-08

**Authors:** Arthur Holbrook

**Affiliations:** Waukesha, Wis.


### ATROPHY OF THE TEETH.

BY ARTHUR HOLBROOK, WAUKESHA, WIS.

Read before the Wisconsin State Dental Society.

Among dentists, an affection of the teeth, characterized either by perforations in, or discolored spots on the enamel, is commonly designated atrophy. The characteristics of genuine atrophy, however, are wanting.

According to modern lexicographers, we find the word applied to express a far different condition. It is defined as "a consumption or wasting of the flesh," "with loss of strength"—"wasting from defective nourishment"—"impaired digestion," etc. The term is not strictly applicable, but as it is evidently the result of altered function and defective nutrition in a portion of one or more of the formative organs, it may be regarded, and is already sanctioned, as the one most convenient for general use.

The affection, more properly considered, would be noticed as a congenital defect, which only occasions a disfigurement, and seldom requires attention from want of proper nourishment, or even from wasting, when the dentine is implicated, and then it is intimately connected with common caries. Only in exceptional cases, when the attack occurs subsequent to the formation of enamel and dentine, is there continual wasting, or any approximation to atrophy.

This exceptional oase, would come under another classification—approaching deundation, which is not in the province of this paper to investigate. We will confine ourselves to the classes enumerated, the spotted and pitted, or grooved, and accept the common appellation.

Odentatrophy, or atrophy of the teeth, has not received the consideration at the hands of the dentist, that its importance demands. Although of less frequent occurrence than most other affections of the teeth, yet it is a bitter infliction when it comes, and the unsightly advertisement of our ignorance, calls for investigation.

Why is it, that we must witness the sorrow and mortification of our patients, and only be permitted to administer a little consolation? Why is it, that we can not tell them the cause, and save those that come after them a double infliction? There are many problems to solve before we can do that, but it is time we commenced a solution of the first.

The Cause.—While other minor affections of the mouth and teeth, are treated with zealous care and minute detail, this sad blight has been entirely overlooked by a majority of dental authors. For instance, Prof. Garretson, in his late work of 700 pages upon the "Diseases of the Teeth and Mouth," devotes about 100 pages to tumors, but not one to atrophy. The "*Cosmos*," one of the oldest and ablest of our dental journals, in all its pages, from the Dental News Letter of 1850, to the last month's number—a space of over twenty years—has not a half dozen articles upon the subject, and of these three fourths are selections from other journals. The hazard of the undertaking is apparent, but there are some that come boldly out. They are like springs in the desert, that strengthen and invigorate the weary, discouraged traveler. Authors like Tomes, of England; Harris, Taft, Judd, and others of our own time, have not hesitated to place themselves upon record. Also, Fox, Maury, Lintoll, Delabarre and others before them, contributed in their time, and from them all, we get the same general conclusions.

Attention will here be called to the accepted theory.

Dental atrophy may be divided into two varieties.

The first variety is characterized by spots, irregular in shape, size and location; varying in color from a pure white to dark-brown, and not confined to any class of teeth. The second variety is distinguished by the well known pitting, or indentations into, and occasionally penetrating the enamel, irregular in shape, location and color; at times connected, forming horizontal grooves. It attacks the incisors more frequently than any of the other teeth, and occurs oftener than the first variety.

A third variety is described by Harris, as a pale, shriveled kind, generally destitute of enamel, and confined to the bicuspids. Although its characteristics are closely allied to the subject, it is not necessary to embrace it here, but press investigation to the second or pitted variety.

The first variety does not impair the uniformity or smoothness of the tooth, and seldom requires attention. It is evidently produced by some cause capable of destroying the bond of union between the enamel and dentine.

"What that cause may be," says one author, "is very obscure."

The second variety does seriously impair the uniformity and smoothness of the tooth, often rendering it unsightly, and causing the keenest anguish. The cause for this variety can be more readily ascertained.

It will not do to rest with the fascinating, easily spoken theory, that it is all the result of a freak of nature, and then dismiss it as we would freckles, warts, or bunions.

Would it not be well to give a little attention to such "freaks of nature," when they are capable of producing such uniform results, and forever manifesting themselves under such peculiar and interesting circumstances?

Among the foremost of modern dentists, and an acknowledged leading anatomist of the day, stands John Tomes, of England. In the American edition of his work on Dental Surgery, page 272, is the following: "Although in many it is not in all cases easy to trace this riged pitted, or honeycombed condition of the teeth to the presence of serious indisposition of the patient, during the time when the defective portions of the tooth were being developed; it can, however, be scarcely doubted that an imperfect formation of the teeth, if not the result of some special disease, such as measles, influencing the system generally, is yet consequent upon a constitutional condition."

"The fact that if one tooth is affected, those parts of other teeth which correspond in respect to the period of formation will present a similar condition, precludes the supposition that the effect has been produced by a merely local cause. The evidence points to a general cause, but it will not uncommonly be very difficult to discover the precise nature of the cause."

Wm. Lintoll, once an eminent English surgeon, in a work published in 1840, says: "The cause of this extraordinary disease is evidently an imperfect formation of the enamel, resulting from disturbance of the system, either by ill health, or by the too free exhibition of mercu y, during the process of secretion by the membrane of the dental sacs." He then cites cases in illustration, from his own observation. Earlier authorities, such as Fox, Maury, Delabarre, and others, made similar expressions, and gave similar views.

Later again, Prof. Harris, writes in his Principles and Practice: "The formative organ of the enamel, as is now generally admitted, consists of a membrane, composed almost wholly of short hexagonal corpuscles or fibres, which correspond in shape and arrangement to the fibres of the enamel. This membrane is moulded accurately to the crown of the tooth, and according to Raschow, each fibre is a secretory duct, whose peculiar function is to secrete the fibre of the enamel corresponding to it.

It should be borne in mind that the secretion of the earthy salts of the enamel, commences at the coronal extremity of the tooth, gradually proceeding toward the base of the crown. Now we can readily conceive that some constitutional disease might interrupt the secretion of the earthy salts deposited in the enamel cells, or secretory ducts of the enamel membrane, for the formation of the enamel fibres, occurring at the time when this process is going on, it might prevent them from being filled, and cause th m to wither or waste away, giving to this portion of the enamel the pitted appearance. In other words, the secretion of the inorganic constituents of the enamel, being interrupted for a short time, the horizontal row of cells in the enamel membrane, into which it should be deposited, will not be filled; consequently, as might be readily supposed, they will waste away, leaving a circular row of indentations around the crown of the tooth. But as soon as the constitutional disease has run its course, the secretion of the earthy salts is resumed, and unless there is a relapse or second attack capable of interrupting the secretory process, the other parts of the enamel will be well formed." * * * He adds: "We believe it almost always occurs as a consequence of some eruptive disease or catarrhal fever, occurring during the process of "enameling," and there are many facts which go to sustain the correctness of this opinion. In nearly all cases that have fallen under our observation, it was clearly traceable to measles, scarlatina, chicken pox, catarrhal fever or small pox. It may, however, be produced by other constitutional diseases." Again: "The occurrence of the constitutional diseases about the time when the affected parts of teeth must have been receiving their earthy salts, would seem to establish, very conclusively, the connection of the one with the other."

Prof. Taft, in his Operative Dentistry, writes: "We are led to infer, then, that the origin of the affection is for the most part constitutional, and not local. There are commonly found traces of it on all the teeth whose enamel was in process of formation at the time of the interruption. Any general disturbance, such as to interrupt the assimilative process, would be detrimental to the perfect formation of the enamel. * * Any disease that would interrupt the functions of the digestive apparatus, would be prejudicial to the process of assimilation—whilst other diseases, such for instance, as those of a fibrile character, would diminish the vital power, and consequently the ability to build up organic structures without interrupting in any special manner the process of assimilation."

In the "*Cosmos*," for last April, will be found an extract from the "*Medical Gazette*," detailing a case reported by

Drs. Perine and Franklin, dentists in New York: "As we are unacquainted with any agents except acids, that are capable of acting upon the enamel or dentine, we are inclined to adopt the theory, that sometime during the continuance of diseased action, the secretions of the body change from alkaline or neutral condition, to an acidulated one, with sufficient solvent powers to either decompose the lime salts before calcific action takes place, causing arrestation of the formative functions, or by attacking the newly formed tooth, and dissolving the lime salts from it. * * * Teeth sometimes present deep pits or grooves across the surface of the enamel, in a more advanced state of development, while those less progressed find immunity."

After enumerating a list of diseases and disorders which would cause the affection, they state that they doubt very much whether any medicine could thus affect the teeth, unless acid remedies might possibly exert a slight action.

This leads us to consider the evidence given. Is it conclusive? Is there a cause? With the testimony before us it can hardly be denied. It can not be traced to a specific cause in all cases, but that is no argument that there is no cause.

There are peculiar conditions that prevent a complete analysis. We have first the want of positive knowledge. Parents and children are mortals, and their testimony can only be taken, when actual observation and experience are devoted to the interests of the teeth in question. The diseases and mishaps of childhood are numerous, and to enumerate them years after, and faithfully describe their peculiarities, from the memory alone, would be almost impossible. There are again, cases that are reliable, upon which we may depend; cases that have peculiar associations in connection with them, to assist the memory, or cases that are not distant or remote from the time, and which the dental adviser may have noticed. Many points are necessary to be considered, in arranging a complete investigation of the cause of this affection, and we have confidence in the ability of those who have expressed themselves, to accept their solution.

That disease and medicine do affect the other members of the human body, often arresting development, or disturbing them from their natural growth, is not denied.

Then why not arrest the development of tooth structure? It is the source of greatest trouble after development, and decaying teeth stare us from every mouth. When we acknowledge our ignorance upon this point, can we sustain our conclusions that disease affects the teeth at all?

It seems plausible, that it is a rule that will apply very closely in both cases.

Any action of medicine or disease. any disturbance of the normal functions of the system, mechanical or physical, subsequent to the formation of the tooth, can and does influence the development of the tooth structure, as well as other parts of the system, and when occurring at that critical period will produce atrophy. When occurring at a later period it will influence the tooth itself, weakening it, penetrating it, and causing disease or common caries.

Upon the development of the teeth, the following from Kolliker's Microscopical Anatomy, American edition page 486: "The first step is the formation of a little scale of dentine upon the apex of the pulp; in the molar teeth there are several of these scales, corresponding with the elevation of the pulp, but they soon coalesce. Immediately after the appearance of this dentinal scale, a thin layer of enamel is deposited from the so called enamel organ upon the roof of the sac, and which coalescing with the dentine, forms the first rudiment of the crown of the teeth. The scale of dentine extends over the pulp and becomes thicker, so that it soon rests upon the pulp like a cap, and finally forms a sort of capsule for it, which as ossification proceeds, and the pulp diminishes, closely and completely embraces it; the deposition of enamel goes on simultaneously with it so that it proceeds from the entire surface of the enamel organ, and becomes more and more considerable. In this manner the whole enamel is eventually deposited around the dentinal layer of the crown, until the former is represented only by a delicate membrane; and the latter presents similar relations to that of the perfect tooth. As yet, there exists no trace of root or cement, they are not formed until the crown is nearly completed, and the tooth is about to emerge." "Their ossification begins (permanent teeth) somewhat antecedent to birth in the first molar, extends in the first, second and third years, to the incisors, canines, and premolars, and finally reaches the second molar." To note the manner, how, and where it commences, and the time, are points of vital interest in this matter. Of course, the time given, is the estimate for another country, and we well know that it varies greatly with this. The periods of ossification also vary in the same country—in the same families—as well as the time for teething, for the tooth is not projected until the root has commenced ossification. Hence in tracing causes we take into consideration the time of ossification, and the time of teething. The period for eruption should correspond in all normal cases, with the period of ossification. Then with the actual facts we may determine the time, and attempt conclusions, for our investigations in that direction are quite uncertain; none of us infallible, and no council of interested parents, children, or friends, can make us so.

At our last meeting, the injurious effects of vaccination were advanced, at the period when it is so critical, in the future, growth, and beauty of the teeth, and a case of unusual interest in support of the theory, was cited.

Prof. Judd, of St. Louis, referred to it in the following extract from a private letter: *"The case you report is an

* Child, age 7; vaccinated, 3; scarlatina, 4; erupted at 5. The distance from edge to 1st line and 2d line equal, about two lines separate; and the distance from 2d line to gum, a trifle more; on central edge only affected of molars.

interesting one, and worthy of study. * * In the first place, it may be taken for granted, that the pits were not formed in the enamel after calcification of the latter was completed, so that if the child was vaccinated after the teeth should have been calcified at that place when the pits appear, it would be a very strong argument in favor of the supposition that there was no connection between the vaccination and the appearance of the pits or defects, and *vice versa*, I think it quite probable that in the case cited, there was a direct connection between the vaccination and the pits." He also adds his testimony to the morbid influences upon the teeth from diseases of childhood, in arresting development, and causing the affection.

The growth of structure is from the ceronal surface, from the cutting edge, which easily accounts for a succession of grooves or pits.

